# Longitudinal changes in intracardiac repolarization lability in patients with implantable cardioverter-defibrillator

**DOI:** 10.3389/fphys.2013.00208

**Published:** 2013-08-12

**Authors:** Abhilash Guduru, Jason Lansdown, Daniil Chernichenko, Ronald D. Berger, Larisa G. Tereshchenko

**Affiliations:** ^1^Whiting School of Engineering, Department of Biomedical Engineering, The Johns Hopkins UniversityBaltimore, MD, USA; ^2^Department of Biology, The Johns Hopkins UniversityBaltimore, MD, USA; ^3^Division of Cardiology, Department of Medicine, Johns Hopkins HospitalBaltimore, MD, USA; ^4^Cardiovascular Division, Department of Medicine, Washington University School of MedicineSt. Louis, MO, USA

**Keywords:** intracardiac electrograms, repolarization lability, ventricular tachyarrhythmia, longitudinal analysis, QT variability index

## Abstract

**Background:** While it is known that elevated baseline intracardiac repolarization lability is associated with the risk of fast ventricular tachycardia (FVT)/ventricular fibrillation (VF), the effect of its longitudinal changes on the risk of FVT/VF is unknown.

**Methods and Results:** Near-field (NF) right ventricular (RV) intracardiac electrograms (EGMs) were recorded every 3–6 months at rest in 248 patients with structural heart disease [mean age 61.2 ± 13.3; 185(75%) male; 162(65.3%) ischemic cardiomyopathy] and implanted cardioverter-defibrillator (ICD) or cardiac resynchronization therapy defibrillator (CRT-D) [201 (81%) primary prevention]. Intracardiac beat-to-beat QT variability index (QTVI_NF_) was measured on NF RV EGM. During the first study phase (median 18 months), participants made on average 2.4 visits. Then remote follow-up was continued for an additional median period of 3 years. Average QTVI_NF_ did not change during the first year after ICD implantation (−0.342 ± 0.603 at baseline vs. −0.262 ± 0.552 at 6 months vs. −0.334 ± 0.603 at 12 months); however, it decreased thereafter (−0.510 ± 0.603 at 18 months; *P* = 0.042). Adjusted population-averaged GEE model showed that the odds of developing FVT/VF increased by 75% for each 1 unit increase in QTVI_NF_. (OR 1.75 [95%CI 1.05–2.92]; *P* = 0.031). However, individual patient–specific QTVI_NF_ trends (increasing, decreasing, flat) varied from patient to patient. For a given patient, the odds of developing FVT/VF were not associated with increasing or decreasing QTVI_NF_ over time [OR 1.27; (95%CI 0.05–30.10); *P* = 0.881].

**Conclusion:** While on average the odds of FVT/VF increased with an increase in QTVI_NF_, patient-specific longitudinal trends in QTVI_NF_ did not affect the odds of FVT/VF.

Augmented intracardiac repolarization lability predicts ventricular arrhythmia (Haigney et al., 2004; Couderc et al., 2007; Tereshchenko et al., 2009), sudden cardiac death (SCD) (Piccirillo et al., 2007) and cardiovascular mortality (Tereshchenko et al., 2012) in patients with structural heart disease and long QT syndrome (Hinterseer et al., 2008). Underlying mechanisms of increased repolarization lability are associated with arrhythmogenic substrate [scar, fibrosis, local heterogeneities in action potential duration and morphology, cell-to-cell uncoupling, stochastic gating of ion channels (Pueyo et al., 2011), and increased sympathetic tone in the ventricles of the heart (Baumert et al., 2011)]. It is known that arrhythmogenic substrate does change over time. However, little is known about neither the rate of changes in repolarization lability over 6–12–18 month period nor the predictive value of these changes.

Dynamic time-dependent association between alternating repolarization variability and cardiac death has been previously reported. The Alternans Before Cardioverter Defibrillator (ABCD) Trial showed that baseline microvolt T-wave alternans (TWA) was significantly associated with sustained ventricular tachycardia (VT)/ ventricular fibrillation (VF) or SCD at 6 months, but lost association at 12 months of follow-up (Costantini et al., 2009). ABCD trial results brought up discussion regarding the necessity for establishing the “expiration date” for TWA results, and raised the question about possible utility of repetitive TWA assessment. While elevated repolarization lability, TWA and other non-invasive risk markers (including decreased heart rate variability) have been found to be associated with life-threatening VT/VF and SCD, none of them are routinely used for risk stratification of primary prevention implantable cardioverter defibrillator (ICD) (Tereshchenko and Berger, 2012). Repeated measurement of risk markers might strengthen their predictive value. However, studies of longitudinal changes in repolarization lability are limited.

Earlier we showed that repolarization lability is present throughout the myocardium (Tereshchenko et al., 2009). We demonstrated that the single baseline measurement of intracardiac QT variability index (QTVI_NF_), was associated with fast ventricular tachycardia (FVT)/VF during the subsequent 16 months of follow-up. However, the effect of longitudinal changes in intracardiac repolarization lability on the risk of FVT/VF is unknown. The goal of the present study was to determine if patients with increasing intracardiac QTVI_NF_ over time experience a greater probability of having FVT/VF than those with decreasing or stable intracardiac QTVI_NF_.

## Methods

The ICD-EGMs study (Tereshchenko et al., 2009) protocol was approved by the Johns Hopkins University and the Washington University Human Studies Committees and all participants gave written informed consent before entering the study.

### Study population

The ICD-EGMs study design (NCT00916435) was previously described (Tereshchenko et al., 2009). Briefly, patients with structural heart disease of either sex older than 18 years were enrolled in the study if they had a transvenous ICD or a cardiac resynchronization therapy defibrillator (CRT-D) device implanted for primary or secondary prevention of SCD. In this study we included only participants who (1) had ICD or CRT-D implanted 1 week before enrollment, (2) made at least 2 consecutive office follow-up visits during the first study phase (2005–2007), (3) had at least 2 analyzable digital recordings of intracardiac EGMs at rest that were at least 30 days apart, (4) had EGMs recorded in sinus rhythm, with (5) identical type of ventricular activation [either ventricular-sensed (VS), or ventricular-paced (VP)] at baseline and at all follow-up visits.

### Recording of intracardiac EGMs and follow-up of study participants

Recording of intracardiac EGMs was performed at every office follow-up visit (every 3–6 months) during the first 2 years of the study (2005–2007). Patients were then followed remotely via Carelink® and Latitude® during next 4 years (2007–2011). Near-field (NF) and far-field (FF) RV intracardiac EGMs were recorded at rest for 5–15 min simultaneously with surface electrocardiogram (ECG) via ICD programmer as previously described (Tereshchenko et al., 2009).

Duration of time periods between follow-up visits varied from patient to patient, due to the observational nature of the study. In order to standardize assessment of longitudinal changes in EGM parameters, we categorized follow-up periods as the following. All follow-up visits that occurred in a period of 1–6 months after the baseline (Visit 1) were considered as Visit 2. Visit 3 EGM recording was performed at any time in a period of 181–365 days after baseline EGM recording. Visit 4 was performed during the 1st half of the 2nd year of follow-up.

### Intracardiac repolarization lability analysis

Intracardiac repolarization lability was measured on NF RV EGM as previously described (Tereshchenko et al., 2009) by Berger's method (Berger et al., 1997), using customized MATLAB (MathWorks, Inc., Natick, MA) software. The R-wave peak was automatically detected on NF EGM channel. Two investigators (AG, JL) defined an intracardiac intervals template by selecting the beginning and the end of major NF EGM deflection, and the end of the T wave. The algorithm then determined how much the repolarization segment of each beat had to be stretched or compressed to match the template. Appropriate selection of fiducial points was verified by the third investigator (LGT). Premature ventricular and atrial beats with one post-ectopic sinus beat were excluded from the analysis. Recordings with more than 15% of ectopic, or noise-distorted beats were excluded.

### Endpoints

Appropriate ICD shocks for FVT/VF served as the endpoints in this study. Programming of the ICD device was based on clinical evaluation of the attending electrophysiologist. ICD interrogation data was adjudicated by an endpoint committee composed of 3 members. ICD therapy occurring for VT or VF was classified as appropriate (Tereshchenko et al., 2009). FVT/VF was defined as VT/VF with an average cycle length (CL) ≤ 240 ms. After the 1st FVT/VF event follow-up was continued, and all subsequent sustained FVT/VF events with appropriate ICD shocks were included in the analysis as the study endpoints.

### Statistical analysis

The association of baseline clinical characteristics and ECG parameters with the type of presenting rhythm was measured by the χ^2^-tests and ANOVA or *t*-test, respectively, for categorical and continuous variables with normal distribution. A test of equality of medians or Wilcoxon rank-sum test was used in case the distribution of parameter was not normal. A *P*-value of <0.05 was considered significant. Data analysis was performed using STATA 12.1 (StataCorp LP, College Station, TX).

As office visits were unequally spaced in time, QTVI variogram was explored, and follow-up time periods were equalized with incremental 6 months-intervals. Possible effects of VP on longitudinal QTVI_NF_ changes were explored in patients with different devices types (single-chamber ICD, dual-chamber ICD, CRT-D), as well as in patients who predominantly (at least 99% of follow-up time and 100% of recorded EGM) had VS, in comparison to VP rhythm. “Spaghetti” plots were examined to study variations of QTVI_NF_ across time for each individual. Smoothed plots were used to explore group response of QTVI_NF_ as a function of time, to study variations of QTVI_NF_ across different individuals. Average changes in QTVI_NF_ per 6 months of follow-up were determined. Patterns of QTVI_NF_ changes over time in patients with and without FVT/VF were compared. Since repeated measures made on the same subject are correlated, within-person correlations matrix of QTVI_NF_ across multiple visits was estimated, and the correlation structure was described. The correlation structure for the residuals was explored after removing the mean trend effect. To standardize QTVI_NF_ at each visit, we subtracted observations in each category (each visit) by the mean for that visit and then divided them by the standard deviation for that visit. Further longitudinal regression analyses took into account the visit-to-visit QTVI_NF_ correlation structure to obtain valid inferences.

We compared the association of FVT/VF events with preceding longitudinal QTVI_NF_ changes in an average study participant (in the population-averaged model), and in specific study subject (in the subject-specific model). Population-averaged marginal model accounting for correlation structure [Generalized Estimating Equations (GEE) model] was developed. Multivariate GEE model was used to determine an association between a population-averaged longitudinal QTVI_NF_ changes, and a subsequent outcome. FVT/VF events with appropriate ICD shocks that occurred after respective QTVI_NF_ measurements served as an outcome. GEE model was adjusted by age, sex, race, left ventricular ejection fraction (LVEF), New York Heart Association (NYHA) heart failure class (dichotomized as NYHA = II), indication for ICD (primary or secondary prevention of SCD), type of cardiomyopathy (ischemic or non-ischemic), history of revascularization procedure (either PCI or CABG), type of implanted device(single-chamber ICD, dual-chamber ICD, CRT-D), type of analyzed rhythm (VS or VP), and use of class III anti-arrhythmic medications.

In order to determine the patient-specific association of longitudinal QTVI_NF_ changes with subsequent FVT/VF, we ran a random intercept model for FVT/VF. We accounted for correlation of the repeated QTVI_NF_ observations by including a random intercept for each patient and control for patient's QTVI_NF_ (centered). Adequate fitting of the model was checked to ensure that the specified quadrature has adequately approximated the likelihood.

## Results

### Study population

**Figure 1:**
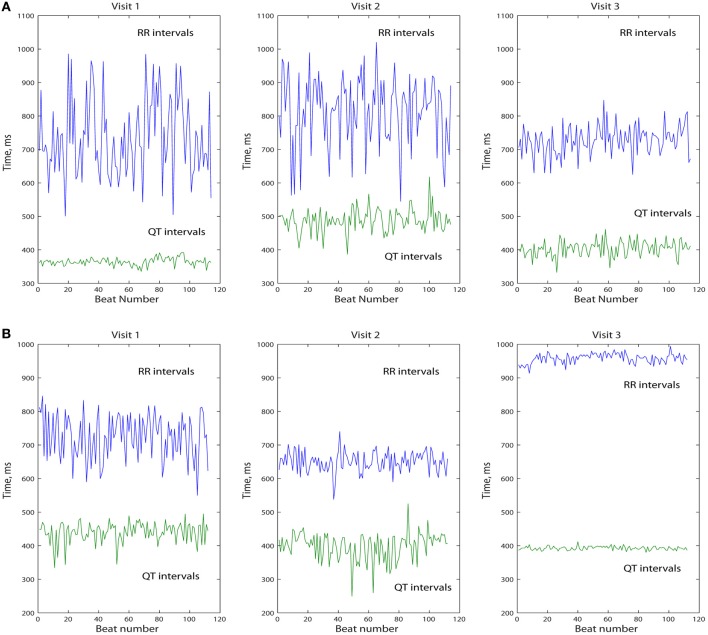
**Representative example of longitudinal changes in beat-to-beat intracardiac QT and RR' variability in patient with (A) and without (B) sustained FVT/VF and appropriate ICD shocks**.

We analyzed the data of 248 study participants: 185 (74.6%) men, 201 Whites (81%), mean age 61.2 ± 13.3 y. Clinical characteristics of study participants are shown in Table 1. We categorized study participants based on their presenting rhythm during follow-up office visits. VS participants were paced <1% during the study duration (Table 1) and were not paced from ventricle(s) during study EGM recordings. Patients who presented with a sinus rhythm, and were 100% VP during the study EGM recordings, comprised VP group. Figure 1 shows representative examples of longitudinal changes in RR' and QT variability in patients with and without ventricular arrhythmia.

**Table 1:** **Comparison of demographic and clinical characteristics of patients with single-, dual-chamber ICD, and CRT-D, and in patients with sinus ventricular-sensed and ventricular-paced rhythm**.

	**Single chamber ICD (*n* = 139)**	**Dual chamber ICD (*n* = 97)**	**CRT-D (*n* = 12)**	**ANOVA P**	**V-sensed (*n* = 195)**	**V-paced (*n* = 53)**	***P*-value**
Age(SD), y	58.2 (13.0)	64.5 (13.3)	69.8 (7.5)	0.0001	60.3 (13.4)	64.5 (12.6)	0.036
Males, *n*(%)	101 (72.7)	74 (76.3)	10 (83.3)	0.636	145 (74.4)	40 (75.5)	0.869
African American, *n*(%)	35 (25.2)	10 (10.3)	2 (16.7)	0.016	40 (20.5)	7 (13.2)	0.229
CHF NYHA class = II, *n*(%)	92 (66.2)	52 (53.6)	12 (100.0)	0.003	118 (60.5)	38 (71.7)	0.135
Ischemic CM, *n*(%)	85 (61.2)	69 (28.9)	4 (33.3)	0.283	70 (35.9)	16 (30.2)	0.439
Primary prevention of SCD, *n*(%)	28 (20.1)	19 (19.6)	0	0.228	157 (80.5)	44 (83.0)	0.680
Presenting VP rhythm, *n*(%)	2 (1.4)	40 (41.2)	11 (91.7)	<0.0001	–	–	–
Atrial pacing %, median(IQR)	–	11.5 (0–76)	0 (0–0)	<0.0001	16 (0–76)	1.5 (0–74)	0.328
Ventricular pacing %, median(IQR)	0 (0–0)	4.5 (0–29.5)	100 (98–100)	<0.0001	0 (0–1)	16 (1–91)	<0.0001
LVEF(SD), (%)	31.9 (12.2)	32.4 (11.3)	25.0 (12.4)	0.127	32.4 (12.1)	29.3 (11.2)	0.075
Diabetes mellitus Hx, *n*(%)	50 (36.0)	23 (24.0)	3 (25.0)	0.132	65 (33.5)	11 (20.8)	0.075
Hypertension Hx, *n*(%)	108 (77.7)	67 (69.8)	10 (83.3)	0.306	150 (77.3)	35 (66.0)	0.093
CABG or PTCA Hx, *n*(%)	65 (46.8)	51 (52.6)	7 (58.3)	0.561	94 (48.2)	29 (54.7)	0.400
History of AF, *n*(%)	32 (23.0)	43 (44.3)	7 (58.3)	<0.0001	55 (28.2)	27 (50.9)	0.002
Beta blockers, *n*(%)	126 (90.7)	78 (80.4)	8 (72.7)	0.038	171 (87.7)	41 (78.9)	0.104
ACE-Inhibitors or ARBs, *n*(%)	111 (79.9)	78 (80.4)	11 (91.7)	0.609	155 (79.5)	45 (84.9)	0.376
Digitalis, *n*(%)	45 (32.4)	39 (40.2)	5 (45.5)	0.375	69 (35.4)	20 (38.5)	0.681
Statin, *n*(%)	97 (69.8)	68 (70.1)	8 (72.7)	0.979	137 (70.3)	36 (69.2)	0.886
Nitrates, *n*(%)	36 (26.1)	20 (20.6)	5 (45.5)	0.170	50 (25.8)	11 (21.2)	0.493
Aldosterone antagonists, *n*(%)	53 (38.1)	37 (38.1)	5 (45.4)	0.888	76 (39.0)	19 (36.5)	0.748
Antidepressants, *n*(%)	30 (21.6)	33 (34.0)	3 (27.3)	0.105	47 (24.1)	19 (36.5)	0.072
Class III antiarrhythmics, *n*(%)	24 (17.3)	33 (34.0)	5 (45.5)	0.004	42 (21.5)	20 (38.5)	0.012

As expected, due to the differences in HF severity, CRT-D patients had significantly higher mean heart rate as compared to single-and dual-chamber ICD patients (Table 2). However, QT interval on NF RV EGM (QT_NF_) was longer in patients with dual-chamber ICDs as compared to those with single chamber ICDs and CRT-Ds. There were no significant differences in heart rate variance, QT_NF_ variance, and QTVI_NF_ amongst patients with different device types, nor in patients with presenting VS vs. those with VP sinus rhythm (Table 2).

**Table 2:** **Comparison of all-visits averaged ECG parameters in patients with single-, dual-chamber ICD, and CRT-D, and in patients with sinus ventricular-sensed and ventricular-paced rhythm**.

	**Single chamber ICD (*n* = 332)**	**Dual chamber ICD (*n* = 230)**	**CRT-D (*n* = 24)**	**ANOVA P**	**V-sensed (*n* = 464)**	**V-paced (*n* = 122)**	***P*-value**
Heart rate(SD), bpm	74.2 (14.5)	71.1 (13.3)	78 (17.5)	0.009	72.8 (14.3)	74.5 (14.2)	0.231
QT interval(SD), ms	469 (116)	496 (110)	478 (180)	0.030	479 (116)	487 (123)	0.477
Heart rate variance, median(IQR), ms^2^	40.2 (7.9–91.5)	47.1 (12.8–99.4)	45.7 (14.0–117.7)	0.549	41.0 (9.1–93.2)	48.6 (13.0–106.7)	0.209
QT variance, median(IQR), ms^2^	748 (122–1693)	729 (167–1704)	556 (269–1190)	0.699	726 (130–1699)	715 (196–1678)	0.617
QTVI(SD)	−0.307 (0.548)	−0.402 (0.648)	−0.293 (0.641)	0.163	−0.344 (0.570)	−0.345 (0.679)	0.983

### Ventricular tachyarrhythmia during follow-up

During the first study phase participants made on average 2.4 visits. FVT/VF events with appropriate ICD shocks were diagnosed in 26 (10.5%) patients. Multiple FVT/VF during follow-up were observed in 20 (77%) out of 26 patients, frequently in clusters. Baseline clinical characteristics in patients with or without FVT/VF during follow-up did not differ (Table 3). Importantly, QTVI_NF_ was the only ECG parameter that differentiated patients with and without arrhythmia. QTVI_NF_ was significantly higher in patients with FVT/VF than in patients without FVT/VF (Table 4).

**Table 3:** **Comparison of baseline clinical and demographic characteristics in patients with and without FVT/VF during follow-up**.

	**No FVT/VF (*n* = 222)**	**FVT/VF (*n* = 26)**	***P*-value**
Age(SD), y	61.9 (12.8)	55.3 (16.6)	0.058
Males, *n*(%)	165 (74.3)	20 (76.9)	0.773
African American, *n*(%)	43 (19.4)	4 (15.4)	0.624
CHF NYHA class = II, *n*(%)	138 (62.3)	18 (69.2)	0.480
Ischemic CM with MI history, *n*(%)	145 (65.3)	17 (65.4)	0.994
Primary prevention of SCD, *n*(%)	180 (81.1)	21 (80.8)	0.969
Single-chamber ICD, *n*(%)	124 (55.9)	15 (57.7)	0.963
Dual-chamber ICD, *n*(%)	87 (39.2)	10 (38.5)	0.963
Bi-Ventricular ICD, *n*(%)	11 (5.0)	1 (3.9)	0.963
Atrial pacing^1^ %, median(IQR)	9.5 (0–71.5)	32 (1–92)	0.053
Ventricular pacing^2^ %, median(IQR)	0 (0–3)	0 (0–0)	0.152
LVEF(SD), (%)	31.7 (11.8)	32.5 (13.5)	0.777
Diabetes mellitus, *n*(%)	68 (30.8)	8 (30.8)	1.00
Hypertension, *n*(%)	165 (74.7)	20 (76.9)	0.901
CABG or PTCA, *n*(%)	111 (50.0)	12 (46.2)	0.711
History of AF, *n*(%)	74 (33.3)	8 (30.8)	0.793
Beta blockers, *n*(%)	192 (86.9)	20 (76.9)	0.169
ACE-Inhibitors or ARBs, *n*(%)	181 (81.5)	19 (73.1)	0.302
Digitalis. *n*(%)	76 (34.4)	13 (50.0)	0.117
Statin, *n*(%)	156 (70.6)	17 (65.4)	0.584
Nitrates, *n*(%)	55 (25.0)	6 (23.1)	0.830
Aldosterone antagonists, *n*(%)	86 (38.9)	9 (34.6)	0.670
Antidepressants, *n*(%)	58 (26.2)	8 (30.8)	0.622
Class III antiarrhythmic medication, *n*(%)	55 (24.9)	7 (26.9)	0.821

1 Percentage of atrial pacing during study phase 1 (counters data) in patients with dual-chamber ICD or CRT-D;

2 percentage of ventricular pacing, accordingly, in patients with ICD only (CRT-D excluded).

**Table 4:** **Comparison of all-visits averaged ECG parameters in patients with and without FVT/VF during follow-up**.

	**No FVT/VF (*n* = 525 visits)**	**FVT/VF (*n* = 61 visits)**	***P*–value**
Heart rate(SD), bpm	73.1 (14.0)	73.4 (16.3)	0.899
QT interval(SD), s	0.479 (0.116)	0.498 (0.129)	0.278
Heart rate variance, median(IQR), ms^2^	45.1 (10.4–99.4)	31.9 (12.8–70.4)	0.143
QT variance, median(IQR), ms^2^	697.0 (147.0–1642.3)	1008 (175.0–2022.4)	0.238
QTV (SD)	−0.362 (0.601)	−0.186 (0.512)	0.015

### The mean trend in longitudinal changes of intracardiac repolarization lability

**Figure 2:**
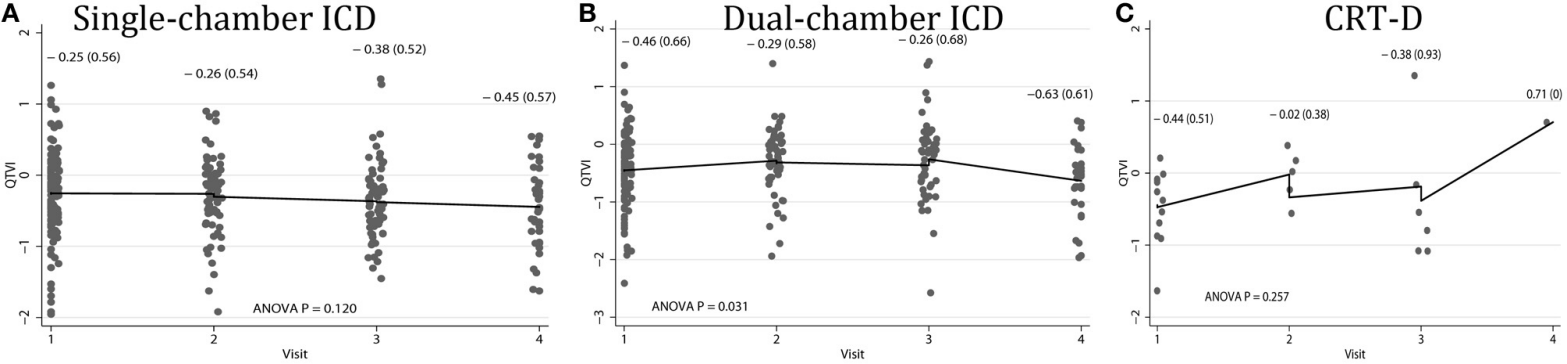
**Comparison of population-averaged longitudinal trends in QTVI_NF_ in patients with (A) single-chamber ICD, (B) dual-chamber ICD, (C) CRT-D.** Scatterplot of QTVI_NF_ at each standardized study visit is shown. Lowess smoother curve shows mean trend in QTVI_NF_ over time.

**Figure 3:**
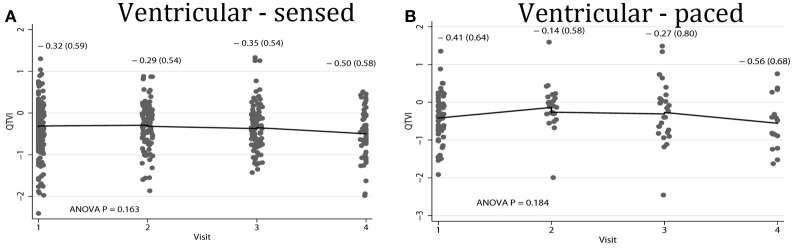
**Comparison of population-averaged longitudinal trends in QTVI_NF_ in patients with (A) presenting ventricular-sensed (VS), and (B) presenting ventricular-paced (VP) sinus rhythm.** Scatterplot of QTVI_NF_ at each standardized study visit is shown. Lowess smoother curve shows mean trend in QTVI_NF_ over time.

**Figure 4:**
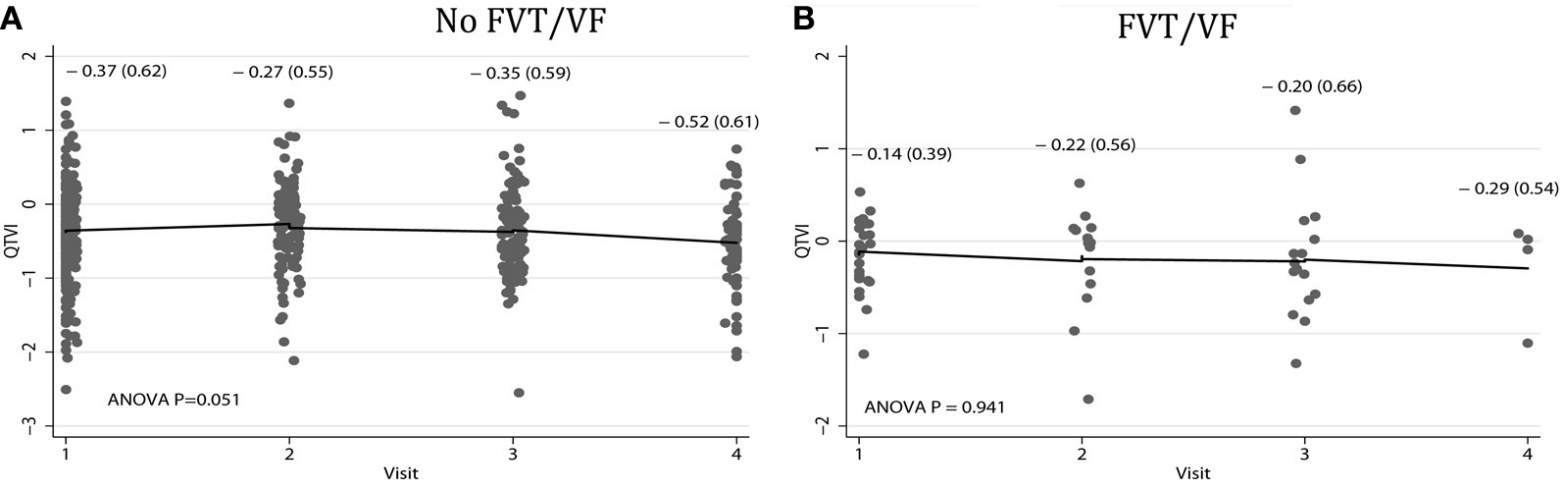
**Comparison of population-averaged longitudinal trends in QTVI_NF_ in patients (A) without FVT/VF, and (B) with sustained FVT/VF and appropriate ICD shocks.** Scatterplot of QTVI_NF_ at each standardized study visit is shown. Lowess smoother curve shows mean trend in QTVI_NF_ over time.

Average QTVI_NF_ in study participants with single-chamber ICD did not change during the study period. However, average QTVI_NF_ in patients with dual-chamber ICDs increased during the first year after ICD implantation, but slightly decreased thereafter (Figure 2). At the same time, no statistically significant differences were observed in all VS, as compared to all VP patients (Figure 3). Averaged trend in QTVI_NF_ was different in patients with FVT/VF, as compared to patients without FVT/VF (Figure 4).

### Subject-specific changes in repolarization lability over time

**Figure 5:**
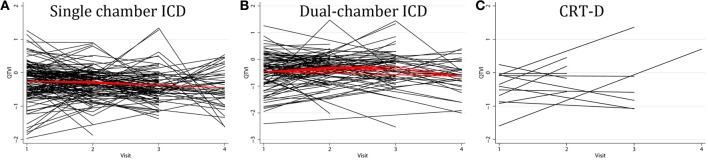
**“Spaghetti” plots of individual patient-specific longitudinal relationships between QTVI_NF_ and time for each subject with (A) single-chamber ICD, (B) dual-chamber ICD, (C) CRT-D**.

**Figure 6:**
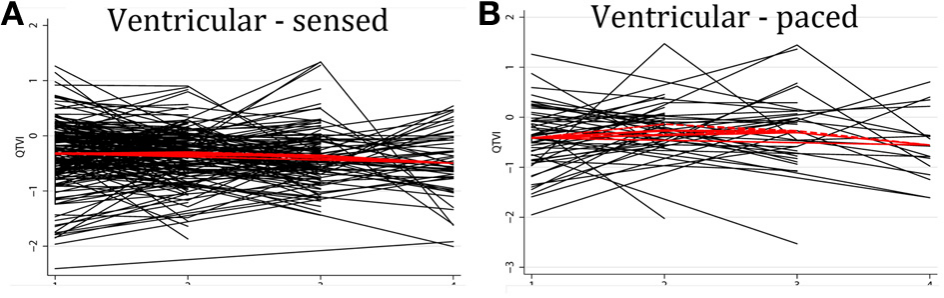
**“Spaghetti” plots of individual patient-specific longitudinal relationships between QTVI_NF_ and time for each subject with (A) presenting ventricular-sensed (VS), and (B) presenting ventricular-paced (VP) sinus rhythm**.

**Figure 7:**
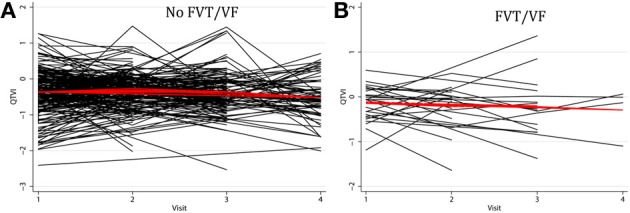
**“Spaghetti” plots of individual patient-specific longitudinal relationships between QTVI_NF_ and time for each subject (A) without FVT/VF, and (B) with sustained FVT/VF and appropriate ICD shocks**.

Individual patients demonstrated drastically different trends: in some patients intracardiac QTVI_NF_ increased while in others QTVI_NF_ decreased, stayed flat, or fluctuated over time. There were no differences in the behavior of the subject-specific longitudinal relationships in patients with different device type (Figure 5), or any between groups of VS and VP patients (Figure 6). Moreover, no differences in subject-specific QTVI_NF_ longitudinal trends were observed in patients with FVT/VF, as compared to patients without FVT/VF (Figure 7). Multiple cross-overs of the lines indicated that the relative order of patients, ordered by their baseline QTVI_NF_, changed over time. The study population-averaged QTVI_NF_ longitudinal relationship (Figures 2–4) was not consistent with the subject-specific longitudinal relationship (Figures 5–7).

We observed weak positive correlation between QTVI_NF_ at the 1st and the 4th visits, and at the 1st and the 2nd visits in patients with single-chamber ICD (*r* = 0.312; *P* = 0.047 and *r* = 0.269; *P* = 0.024, respectively) and VS patients (*r* = 0.297; *P* = 0.026 and *r* = 0.384; *P* < 0.0001, respectively), and weak negative correlation in QTVI_NF_ at the 3rd and 4th visit (*r* = −0.604; *P* = 0.003 in single-chamber ICD group; *r* = −0.426; *P* = 0.021 in VS patients). Thus, in a given patient with single-chamber ICD who did not experience ventricular pacing, QTVI_NF_ 1 week after ICD implantation more likely positively correlated with QTVI_NF_ 1.5 years after ICD implantation. If such a patient experienced elevation of QTVI_NF_ during the 1st year post-ICD implantation, then during the next 6 months QTVI_NF_ was more likely decreasing (negative correlation in QTVI_NF_ between the 3rd and the 4th visits). No significant correlations between QTVI_NF_ observations at different visits in VP patients, both with dual-chamber ICDs and CRT-D devices were found.

### Association between longitudinal changes in repolarization lability and subsequent FVT/VF

The mean QTVI_NF_ trend in patients without FVT/VF demonstrated slight, but significant decrease 1.5 years after device implantation (Figure 4A), whereas no changes in mean QTVI_NF_ were observed in patients with FVT/VF (Figure 4B). Patterns of the subject-specific relationships between QTVI_NF_ and time looked alike in patients with and without FVT/VF (Figures 7A,B). QTVI_NF_ correlations structure in patients without FVT/VF revealed weak positive correlation between the 1st and the 2nd visit (*r* = 0.257; *P* = 0.004) and negative correlation of approximately the same strength between the 3th and the 4th visit (*r* = −0.339; *P* = 0.040). However, QTVI_NF_ measured at different visits in a given patient who experienced FVT/VF during follow-up, did not correlate.

**Figure 8:**
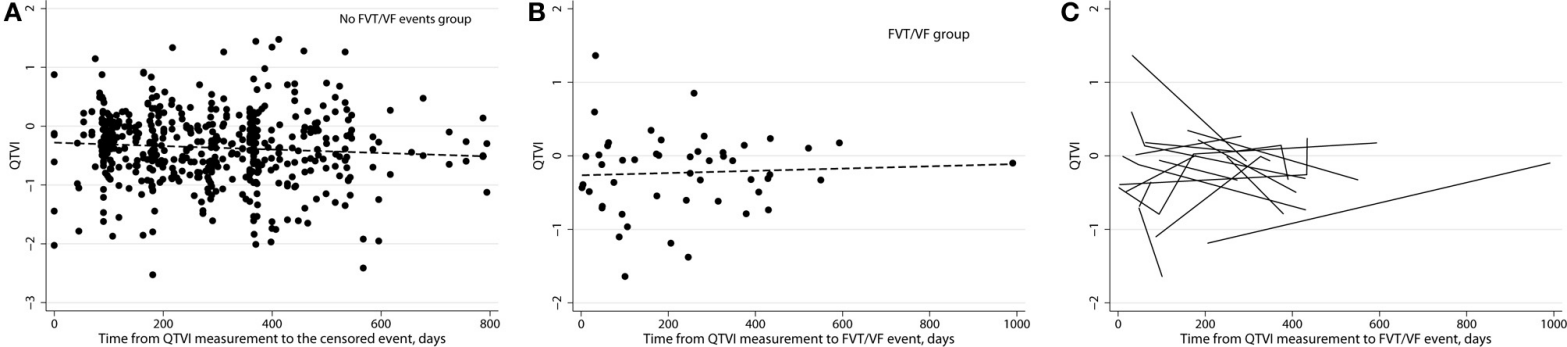
**(A)** Variogram of QTVI_NF_ in patients without FVT/VF shows actual time since EGM recording till censored. **(B)** Variogram of QTVI_NF_ in patients with FVT/VF shows actual time since EGM recording till sustained FVT/VF with ICD shock. **(C)** Line “spaghetti” plots of the longitudinal relationships between QTVI_NF_ and actual time of FVT/VF for each patient with FVT/VF event.

In order to study patient-specific dynamic changes in intracardiac RL before FVT/VF, we plotted QTVI_NF_ before each FVT/VF event. We used the actual time from then EGM recording to the FVT/VF event as a continuous variable (Figure 8). Noticeably, QTVI_NF_ distribution in patients without FVT/VF (Figure 8A) looked similar to that in patients before FVT/VF (Figure 8B). Trends of increasing, decreasing, and flat over time QTVI_NF_ were observed before FVT/VF events (Figure 8C). Of note, consistent pattern of increasing over time QTVI_NF_ before all FVT/VF events was observed in some (but not all) individual patients with multiple FVT/VF events. In contrast, patients with a single FVT/VF event tended to demonstrate rather decreasing over time QTVI_NF_. However, small subgroups size did not allow us to quantify observed differences.

### GEE population-averaged model

In multivariate GEE analysis with independent correlation structure, increasing over a time course of several months QTVI_NF_ was significantly associated with FVT/VF event [OR 1.75 (95%CI 1.05–2.92); *P* = 0.031]. Thus, on average, for patients in our study, the odds of developing FVT/VF increased by 75% for each 1 unit increase in QTVI_NF_.

### Subject-specific random intercept model

Association between patient-specific dynamic changes in QTVI_NF_ and FVT/VF was studied in the random-effects logistic regression. In order to determine if a specific patient with increasing QTVI_NF_ over time experiences a greater probability of having FVT/VF, as compared to patient with decreasing or stable QTVI_NF_, we ran random-effects logistic regression analysis. For a given patient, the odds of developing FVT/VF were not associated with increasing or decreasing QTVI_NF_ [OR 1.27; (95%CI 0.05–30.10); *P* = 0.881] over time. We used 194 integration points in this model for assurance that the likelihood is appropriately approximated, which was confirmed. In addition, we explored potential interactions and adjusted patient-specific models for the effect of VP in preceding follow-up interval, for percentage of beats, included in QTVI analysis by automated software, and for the differences in follow-up time interval. Subject-specific longitudinal QTVI_NF_ trend was associated neither with study outcome (FVT/VF), nor with any other measured in this study clinical, demographic, or ECG parameter.

## Discussion

Our study revealed differences in population-averaged and patient-specific intracardiac QTVI_NF_ trends over 1.5 years after device implantation. In patients without FVT/VF events population-averaged QTVI_NF_ decreased after the 1st year since ICD implantation, whereas in patients with FVT/VF events averaged QTVI_NF_ did not change. Group-averaged QTVI_NF_ was independently associated with the odds of life-threatening FVT/VF. On average, the odds of FVT/VF increased by 75% for each 1 unit increase in QTVI_NF_.In contrast, individual subject-specific QTVI_NF_ trends in many patients differed from the mean trend for the whole study population. Individual subject-specific trends in intracardiac QTVI_NF_ were not associated with subsequent FVT/VF.

### Longitudinal studies of repolarization lability: mean trend vs. subject-specific changes over time

Population-averaged trends in repolarization lability and TWA changes immediately before VT/VF have been previously studied. The vast majority of investigators explored short-term (24–48 h) dynamic ECG changes. Shusterman et al. (2006) described an upsurge of alternating and non-alternating RL 10 min before the onset of VT/VF in HF patients. Analysis of 24-h Holter ECG revealed pronounced diurnal variations in QTVI (higher QT variability in the morning and during the day, and lower QT variability during the night) in HF patients (Dobson et al., 2009). At the same time, no significant changes in the mean QTVI trend was detected during 12 h of ECG monitoring before the onset of spontaneous VT/VF in the acute intensive cardiac care unit patients (Sachdev et al., 2010). In this study population-averaged model reflected absolute difference in QTVI_NF_12–18 month after ICD implantation in patients with and without subsequent FVT/VF (Table 4; Figure 4), and re-confirmed our previous finding of association between elevated QTVI_NF_ and VT/VF (Tereshchenko et al., 2009).

Very few investigators studied subject-specific trends in repolarization lability. Swerdlow and co-investigators (2011) used subject-specific longitudinal analysis approach and showed increased intracardiac TWA/variability immediately before VT/VF onset in ICD patients. To the best of our knowledge, our study is the first that compared patient-specific and population-averaged longitudinal trends in intracardiac QTVI_NF_. Mechanistically, each individual's longitudinal outcome is governed by subject-specific disease dynamics over time. Hence, each subject's repolarization lability trajectory neither necessarily progresses in accord with a rigid population mean curve nor varies with bounded variation. Evidently, some “latent” factors (not measured in this study) impacted individual subject-specific trends in QTVI_NF_. In patients with structural heart disease and systolic HF multiple parameters might change over time: coronary perfusion, myocardial contractility and compliance, use of medications, affecting repolarization (Tereshchenko et al., 2009), level of physical activity and autonomic balance, kidney function, and many others. Any of these unmeasured in this study factors might play a major role in the observed subject-specific QTVI_NF_ changes.

Recently we showed that both high and low repolarization lability is associated with the risk of SCD in general population (Tereshchenko et al., 2012). In our previous analysis of ICD-EGMs study we observed that QTVI_NF_ was decreased after premature ventricular contraction. Paradoxically, decreased, but not increased QTVI_NF_ was associated with VT/VF (Das et al., 2012). While arrhythmogenesis, associated with elevated repolarization lability is well understood (Tereshchenko and Berger, 2011), observation of association between decreasing QTVI_NF_ and subsequent FVT/VF is novel and prompts further investigations. As previously shown, static QT/RR relationships (Batchvarov et al., 2002), as well as the dynamic pattern of QT/RR hysteresis (Malik et al., 2008) is highly patient-specific. It was even suggested that the individual QT-RR relationship has unique “finger-print-like” properties (Malik et al., 2008). This fact might contribute to the high degree inter-subject variability of the dynamic QTVI_NF_ patterns, observed in this study. Especially intriguing was observation of consistent increasing QTVI_NF_ trend before each FVT/VF event in some (but not all) patients with multiple FTV/VF events. Further mechanistic studies are needed to explore possible mechanisms of arrhythmiogenesis, associated with decreasing QTVI_NF_, in order to explain subject-specific trends fully.

### Effect of ventricular pacing on repolarization lability

In this study we did not find significant differences in intracardiac QTVI_NF_ between VS and VP patients in sinus rhythm. This observation is in concordance with our earlier study (Tereshchenko et al., 2011) of intracardiac QTVI_NF_ in CRT patients. Of note, we longitudinally studied only patients with consistently identical type of the presenting rhythm across multiple visits.

Since deleterious effect of RV pacing is well known (Wilkoff et al., 2002), we focused our analysis on evaluation of the possible longitudinal effects of amount of RV pacing on QTVI_NF_ and study outcomes. We did not find significant differences in percentage of VP in patients with and without FVT/VF. Furthermore, the percentage of VP did not influence QTVI_NF_ and was not responsible for fluctuations of QTVI_NF_ from visit-to-visit.

### Clinical perspective

In clinical practice and clinical research multiple physiological parameters are measured repetitively and are studied longitudinally. In the vast majority of longitudinal studies population-averaged analysis is the only analysis applied. Very few longitudinal studies in electrophysiology report results of subject-specific longitudinal analysis. However, the physician manages the specific individual patient, rather than the “population-averaged” patient, and understanding of the patient-specific trends is extremely important for clinical decisions in the era of individualized medicine. In this study, population-averaged and subject-specific longitudinal trend in repolarization lability demonstrated different degree of association with subsequent FVT/VF. Further prospective longitudinal studies of repolarization lability should be conducted in order to understand behavior and predictors of subject-specific longitudinal trends in repolarization lability, and to determine its association with clinically-meaningful outcomes.

### Limitations

Several limitations have to be acknowledged. First of all, the EGM recordings were obtained not exactly at the same time of the day during all follow-up visits. However, previous circadian study of QT variability did not find significant differences in QT variability during the day (Dobson et al., 2009). All EGMs were recorded during the morning and middle-day hours (8 am–3 pm), which minimized possible circadian effect.

Time intervals between follow up visits varied due to the observational nature of the study. In order to overcome this limitation, we standardized time intervals for longitudinal analysis. In addition, we employed specific analytical approach, which incorporated actual time from EGM recording to outcome.

## Funding sources

This study was supported by Medtronic, Inc., as an Investigator-initiated Research Project.

### Conflict of interest statement

This study was supported by Medtronic, Inc., as an Investigator-initiated Research Project. Ronald Berger holds a patent on the technology for QT variability analysis. The other authors declare that the research was conducted in the absence of any commercial or financial relationships that could be construed as a potential conflict of interest.
